# Secretory Phospholipase A_2_-IIA Protein and mRNA Pools in Extracellular Vesicles of Bronchoalveolar Lavage Fluid from Patients with Early Acute Respiratory Distress Syndrome: A New Perception in the Dissemination of Inflammation?

**DOI:** 10.3390/ph13110415

**Published:** 2020-11-23

**Authors:** Stylianos Papadopoulos, Eleftheria Kazepidou, Marianna H. Antonelou, George Leondaritis, Alexia Tsapinou, Vasilios P. Koulouras, Apostolos Avgeropoulos, George Nakos, Marilena E. Lekka

**Affiliations:** 1 Laboratory of Biochemistry, Department of Chemistry, University of Ioannina, 451 10 Ioannina, Greece; st.papadopoulos@uoi.gr (S.P.); elkazep@yahoo.com (E.K.); alexiatsapinou@gmail.com (A.T.); 2 Section of Cell Biology & Biophysics, Department of Biology, School of Science, National and Kapodistrian University of Athens (NKUA), Panepistimioupolis, 15784 Athens, Greece; manton@biol.uoa.gr; 3 Laboratory of Pharmacology, School of Medicine, University of Ioannina, 451 10 Ioannina, Greece; gleondar@uoi.gr; 4 Department of Intensive Care Medicine, School of Medicine, University of Ioannina, 451 10 Ioannina, Greece; vpkoulouras@yahoo.gr (V.P.K.); gnakos@uoi.gr (G.N.); 5 Department of Materials Science, University of Ioannina, 451 10 Ioannina, Greece; aavger@uoi.gr

**Keywords:** phospholipase A_2_, extracellular vesicles, exosomes, bronchoalveolar lavage fluid, acute lung injury, acute respiratory distress syndrome, immuno-TEM

## Abstract

Secretory phospholipase-IIA A_2_ (sPLA_2_-IIA) is expressed in a variety of cell types under inflammatory conditions. Its presence in the bronchoalveolar lavage (BAL) fluid of patients with acute respiratory distress syndrome (ARDS) is associated with the severity of the injury. Exosomal type extracellular vesicles, (EVs), are recognized to perform intercellular communication. They may alter the immune status of recipient target cells through cargo shuttling. In this work, we characterized the exosomal type EVs isolated from BAL fluid of patients with early and late ARDS as compared to control/non-ARDS patients, through morphological (confocal and electron microscopy) and biochemical (dynamic light scattering, qRT-PCR, immunoblotting) approaches. We provide evidence for the presence of an sPLA_2_-IIA-carrying EV pool that coprecipitates with exosomes in the BAL fluid of patients with ARDS. *PLA2G2A* mRNA was present in all the samples, although more prominently expressed in early ARDS. However, the protein was found only in EVs from early phase ARDS. Under both forms, sPLA_2_-IIA might be involved in inflammatory responses of recipient lung cells during ARDS. The perception of the association of sPLA_2_-IIA to the early diagnosis of ARDS or even with a mechanism of development and propagation of lung inflammation can help in the adoption of appropriate and innovative therapeutic strategies.

## 1. Introduction

The phospholipase A_2_ (PLA_2_) superfamily of enzymes catalyze the hydrolysis of ester bonds at the *sn-2* position of glycerophospholipids generating lysophospholipids and free fatty acids. Their physiological roles range from phospholipid biosynthesis/membrane remodeling, where mainly cytosolic types are involved [1], to cell signaling, host defense [2], and to the generation of potent inflammatory mediators, such as eicosanoids and platelet activating factor (PAF) [3]. Secretory and cytosolic PLA_2_ isotypes can act in concert in membrane trafficking and fusion events [4,5,6] and their expression can be either constitutive or inducible. The presence of secretory types of PLA_2_, (sPLA_2_), especially of the IIA isotype, in biological fluids, such as synovial fluid and plasma from patients with arthritis, atherosclerosis, acute lung injury, sepsis and cancer [7], underlines their involvement in inflammatory, autoimmune and allergic diseases. sPLA2-IIA acts as an acute phase protein [8,9], exhibiting catalytic or even non-catalytic functions in host defense [10,11]. Indeed, recent data, suggest that sPLA_2_-IIA binds to integrins and shows alternative functions due to integrin-dependent signaling pathways [12]. Especially in the lung, the levels of the enzyme have been studied in patients with early phase acute respiratory distress syndrome (ARDS) as well as in animal ARDS models (for review, see ref [13]). Our group found elevated levels of sPLA_2_-IIA in the BAL fluid of early-phase ARDS patients, which correlated positively with the clinical severity of the disease [14,15,16]. The early phase ARDS, also called exudative phase, is characterized by acute inflammation, deterioration of the alveolar-capillary membrane barrier and edema formation, while in the late ARDS phase, or recovery phase, fibrotic and restoration events prevail [17]. The profile of the substances and mediators in these two phases are quite distinct. Bronchoalveolar lavage (BAL) fluid, which represents alveolar fluid in approximately 100-fold dilution, is a useful material for the diagnosis of lung disorders; it permits the evaluation of the stage of the disease and the responsiveness of the disease to therapeutic approaches. Under physiological conditions, alveolar macrophages are the most abundant cell population in BAL fluid. However, in the case of inflammation, especially during the acute phase of ARDS, inflammatory cells, including blood monocytes and polymorphonuclear cells, invade from the circulation into the lung, along with various pro-inflammatory and anti-inflammatory agents due to the increased alveolar-capillary permeability [18]. The composition of these cell populations, their activation state, the type of secreted substances and the patient’s outcome depend largely on the type of insult and the time elapsed from the insult [19,20,21,22]. BAL fluid also contains lung surfactant formations, namely, the surface-active phospholipid/protein material that lines the alveoli.

The release of extracellular vesicles (EVs) from cells, in a constitutive or inducible manner, is a ubiquitous process. EVs are present in biological fluids including plasma, urine and alveolar fluid represented in BAL fluid [23]. Their cargo is relevant not only to the cell type, but also to the stimulus that may trigger their release. Among them, dense EVs of endosomal origin, sized from 30 to 150 nm, called exosomes, have emerged as organelles performing intercellular communication. They are generated by inward budding of the endosomal membrane and are, consequently, released in the extracellular space upon fusion with the plasma membrane. They carry specific signals, bioactive lipids, proteins, RNA and microRNAs that can affect the immune status of target cells [24]; however, a direct correlation between the cargo composition, the type of insult and the function of exosomes remains largely unclear.

Although sPLA_2_ type IIA was the first PLA_2_ to be discovered, its secretion mechanism from cells and its function is still not well understood. In this work, we investigated whether sPLA_2_-IIA can be released in the alveolar space by vesicular shedding and whether these vesicles bear size, density and biomarkers relevant to endosome-derived, exosome-type of EVs. In this regard, the study was performed on isolated EVs from the BAL fluid of early, late ARDS and from control, non-ARDS patients.

## 2. Results

### 2.1. Characterization of BAL Fluid Derived EVs

To examine the nature of extracellular vesicles present in the BAL fluid samples, a protocol of isolation via differential centrifugations was followed, as described in Materials and Methods.

EVs of exosomal type typically float at densities ranging from 1.13–1.19 g/mL, although there are reports of exosomal-like EVs isolated from higher density fractions [25]. Table 1 presents the physical characteristics of one representative density gradient fractionation of crude small EVs preparation from BAL fluid. Fractionation took place on a continuous sucrose gradient, while examination of the density fractions by Dynamic Light Scattering (DLS) showed that the vesicles were sized from 30 to 150 nm in diameter. High polydispersity indices for these fractions were observed, suggesting that BAL fluid -isolated vesicles exhibited a significant level of heterogeneity.

**Figure 1:**
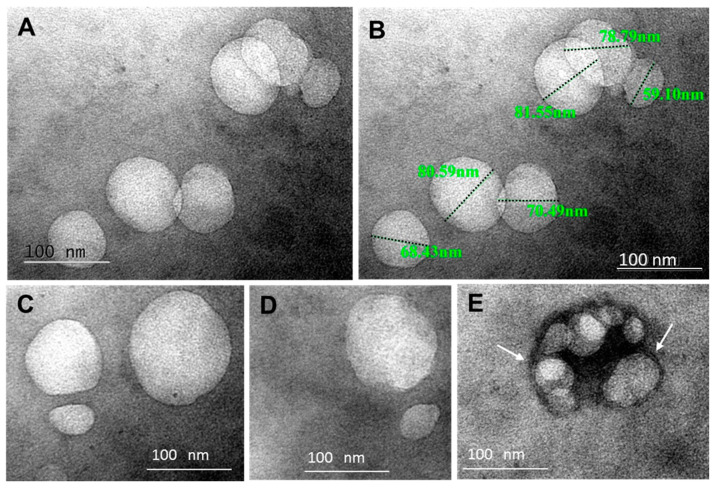
Transmission electron microscopy (TEM) analysis of a crude small extracellular vesicle (EV) pellet from one early ARDS patient’s BAL fluid by the whole mount method. The 120,000 g pellet of the 40,000 g supernatant was fixed with 4% paraformaldehyde and stained with 2% OsO_4_. The majority of the individual EVs were less than 100 nm (**A**,**B**), although EVs of 120–140 nm (**C**,**D**) and multivesicular bodies of small EVs (25–60 nm) enclosed by a lipid bilayer (arrows in **E**) were also detected. The pictures are representative of 9 different BAL fluid isolations.

Detailed morphological analysis of the isolated crude EVs’ fractions from BAL fluid samples was performed by conventional TEM, applying the whole mount method. We observed membrane-surrounded globular or more elliptic and irregular vesicles, in the size-range of “exosomes”, namely of 50 to 100 nm (Figure 1A,B) and some bigger, up to 150 nm (Figure 1C,D). Membrane-enclosed irregular multivesicular body-like structures 160 nm in diameter, filled with packed aggregates of small intralumenal vesicles (25–65 nm), were detected among the individual or grouped vesicles in the pellet (Figure 1E). The sample shown in Figure 1 was obtained from an early ARDS patient, but it is important to point out here that we did not observe any systematic differences in the morphology or density of vesicles isolated from the BAL fluid of patients with or without ARDS. Collectively, these results suggest that BAL fluid contains EVs with morphological characteristics relevant to “exosomes”.

**Figure 2:**
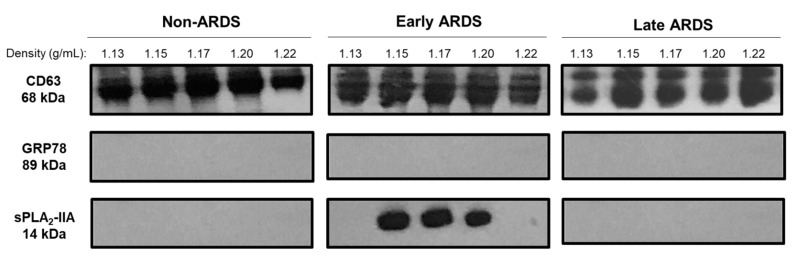
Immunoblotting analysis of CD-63, Grp-78 and sPLA_2_-IIA in BAL fluid density fractions from representative control, early and late ARDS samples. The pictures are representative of 9 early, 5 late ARDS and 7 non-ARDS crude small EVs isolations, fractionated on sucrose density gradient.

We then proceeded to the biochemical characterization of those EVs, testing for the expression of an established exosomal marker, the tetraspanin CD63 in density gradient fractions corresponding to 1.15–1.20 g/mL density range. CD-63 showed a robust expression in the EVs from patients with early or late ARDS and non-ARDS (Figure 2, top panel). Under reducing conditions, typical 50 and 40 kD bands appear with a comparable density among the different groups of patients. In contrast, in these fractions, we could not detect GRP78, a calcium binding protein involved in ER stress, used as a negative marker for exosomes (Figure 2, middle panel). These findings suggest that BAL fluid-isolated small vesicles express one positive marker of exosome-like EVs and that these specific fractions are not contaminated with ER, verifying the specificity of the biochemical isolation.

### 2.2. sPLA_2_-IIA in BAL Fluid-Derived EVs

In previous studies, we showed that total PLA_2_ activity levels were elevated in BAL fluid from early ARDS patients and that this increase was mainly due to secretory PLA_2_ isoforms [13,15]. In the present work, we investigated the probable association of sPLA_2_-IIA with EVs isolated from BAL fluid. After sucrose density gradient centrifugation, we probed for the presence of sPLA_2_-IIA in fractions of representative control, early ARDS and late ARDS samples by immunoblotting. As shown in Figure 2 (bottom panel), the sPLA_2_-IIA was detected in the EVs from patients with early ARDS, but not in EVs from late ARDS or non-ARDS patients. Overall, these data clearly suggest that at least a pool of sPLA_2_-IIA is associated with the EVs isolated from BAL fluid of patients with early ARDS.

**Figure 3:**
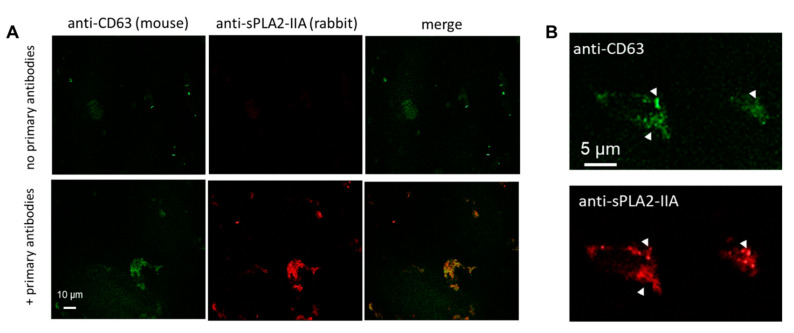
Representative immunofluorescence analysis of crude small EV pellets from one early ARDS patient’s BAL fluid. Vesicles were fixed and stained for the membranous exosomal marker CD63 and sPLA_2_-IIA. (**A**) Control samples in the absence of primary antibodies are shown in the top panel. In the bottom panel, nanovesicle formations presumably aggregates of nanovesicles, were stained positively for both CD63 marker and sPLA_2_-IIA. (**B**) Close examination of stained samples revealed puncta of intense sPLA_2_-IIA staining in CD63 rich aggregates (arrowheads).

To verify the presence of sPLA_2_-IIA in the BAL fluid EVs from patients with early ARDS, we performed a co-staining immunofluorescence experiment. Aliquots from the crude small EV fractions of patients with early ARDS were applied on poly-l-lysine (PLL) coated coverslips, fixed and mildly permeabilized before double immunostaining, to examine the putative co-localization of the exosomal marker CD63 with sPLA_2_-IIA. Specificity of staining was assessed by the omission of primary antibodies, as shown in Figure 3A (top panel). In these experiments, we observed occasional aggregates of vesicles upon incubation on PLL-coated coverslips, the majority of which were co-stained by anti-CD63 and anti-sPLA_2_-IIA antibodies (Figure 3A, bottom panel). Closer examination of stained samples at higher magnification revealed punctas of intense sPLA_2_-IIA staining with single sPLA_2_-IIA spots also positive for CD63 (arrowheads in Figure 3B). Collectively, these results offer additional evidence that sPLA_2_-IIA is physically associated with CD63^+^ exosomal-like BAL fluid EVs of the early ARDS patients, either peripherally or as a cargo.

**Figure 4:**
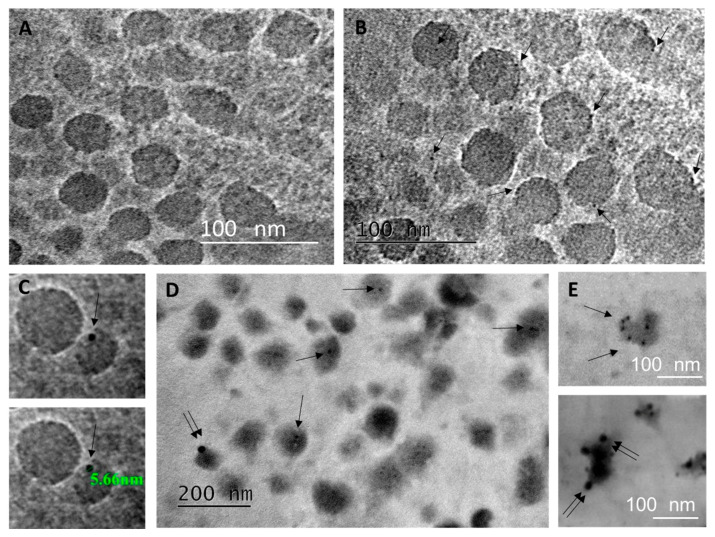
Immunogold electron microscopy of crude small EV pellet from one early ARDS patient’s BAL fluid by the whole mount method. (**A**–**C**) The EV pellet was applied on thin carbon-coated grids, fixed with 4% paraformaldehyde, blocked with BSA 3%, stained with anti-CD63 (in mouse) and detected with anti-mouse IgGs conjugated with 5 nm gold particles and Uranyl Acetate (UA). (**A**) Negative control to show the specificity of the secondary Ab (working dilution of the gold particles; BSA instead of anti-CD63). (**B**) The small EV pellet was positively labeled with the CD63-specific gold particles (arrows). (C) Higher magnification to show the size of the gold particles used. (**D**,**E**) Double immunodetection of sPLA_2_-IIA (anti-Rb IgGs 15 nm gold) in the CD63-positive (anti-Mo IgGs 5nm gold) exosome pellet after saponin permeabilization of the lipid bilayer. CD63-positive EVs (5 nm, single arrows) were detected in the vicinity of sPLA_2_-positive EVs (15 nm, double arrows). (**E**) Higher magnification of individual EVs showing heavy decoration with CD63 or sPLA_2_-specific gold particles.

To further investigate whether sPLA_2_-IIA might be a bonafide cargo of EVs isolated from BAL fluid of patients with early ARDS, we then proceeded with whole mount immuno-electron microscopy experiments. EVs were single immunolabeled with antibody against CD63 (Figure 4B,C), or double immunolabeled for CD63 and sPLA_2_-IIA, following a membrane permeabilization step (Figure 4D,E). Specificity of staining was assessed by routine procedures, including omission of primary antibodies (Figure 4A). Gold particles of 5 nm (Figure 4C) representing CD63 exosome marker were detected in many EVs in single and double immunolabelings (single arrows in Figure 4B–E). The bigger gold particles of 15 nm, representing sPLA_2_-IIA, were less common but occasionally there was a heavy decoration on individual EVs (Figure 4E), as in the case of CD63, suggesting that sPLA_2_-IIA may be a component of early ARDS BAL fluid vesicles. Co-localization of CD63 and sPLA_2_-IIA was also detected, though in fewer EVs of loose structure, probably due to the weak staining for sPLA_2_-IIA and the detrimental effect of membrane permeabilization step that is hardly controlled in exosomal membranes.

### 2.3. sPLA_2_-IIA mRNA Content in BAL Fluid-Derived EVs Is Increased in Early ARDS Patients

Having shown that BAL fluid of early ARDS patients is characterized by a pool of sPLA_2_-IIA molecules associated with exosomal type EVs, we investigated whether sPLA_2_-IIA mRNA is also present in this fraction, since EVs are well established vehicles for long-range delivery of mRNAs across tissues and cells. We thus measured the levels of *PLA2G2A* (gene name corresponding to sPLA_2_-IIA) mRNA in EVs isolated from BAL fluid of early, late ARDS and non-ARDS patients with qRT-PCR.

**Figure 5:**
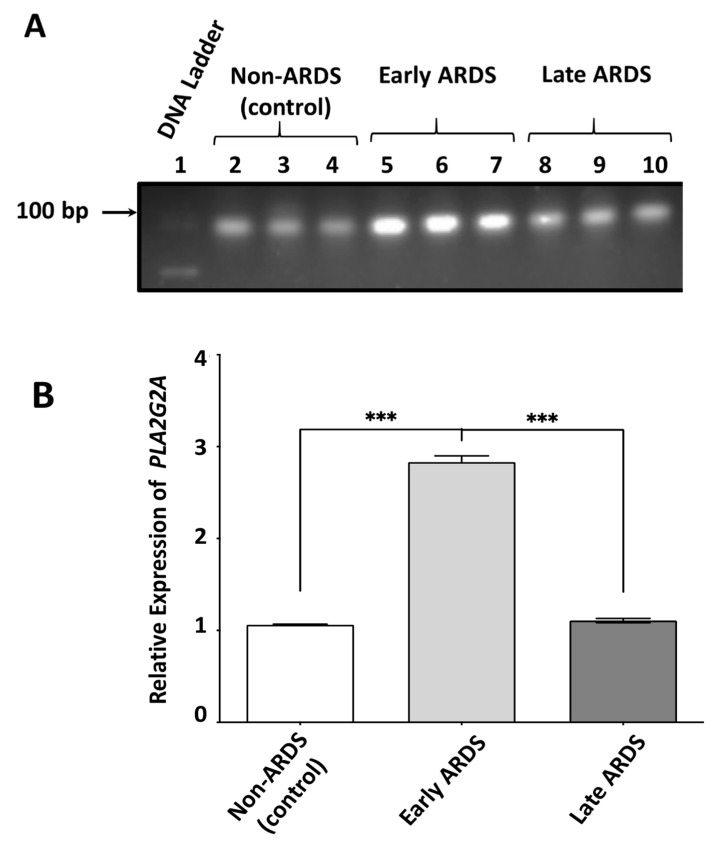
Determination of *PLA2G2A* in BAL fluid’s exosomal type EVs by quantitative real-time PCR. Exosomal type EVs were isolated and purified from BAL fluids of 9 patients with early phase ARDS, 5 late phase ARDS and 7 non-ARDS-control subjects. (**A**) Electrophoretic analysis of qRT-PCR (cDNA) products using 2% agarose gel. The *PLA2G2A*-specific band was identified at 86 bp. Pictures show representative mRNA isolations from three different patients per group. (**B**) Relative expression of *PLA2G2A* in EVs from BAL fluid. The expression levels of *PLA2G2A* were normalized to those of *GAPDH*. The data represent the Means ± SE analyzed in duplicates. *** *p* < 0.001: statistical significance as assessed by one-way ANOVA test.

Our experiments showed that *PLA2G2A* mRNA is present in EVs from both control and ARDS patients (Figure 5A). Interestingly, *PLA2G2A* mRNA levels from EVs of early ARDS patients were approximately three times greater than those of both control non-ARDS patients [(1.77 ± 0.12) -fold] and of late ARDS patients [(1.72 ± 0.08)] -fold, (Figure 5A,B). We did not find any significant difference in the *PLA2G2A* mRNA content of EVs from control and late ARDS patients. Collectively, our results suggest that the crude small EVs in the BAL fluid from patients with early ARDS, that contain sPLA_2_-IIA protein, are further enriched with *PLA2G2A* mRNA.

## 3. Discussion

Lung inflammation can be induced through the continuous exposure of airways to harmful environmental substances, although systemic dissemination can also occur [26,27]. Moreover, physical forces developed during mechanical ventilation can maintain certain levels of inflammatory response [28]. In normal lung, resident alveolar macrophages, representing the main cellular population in the alveoli, are in the first line of lung defense. In the case, however, of acute lung injury and its severe form, ARDS, pro-inflammatory cells are recruited in the alveoli and secrete different mediators to confront the injury [21,29]. Among them, various types of phospholipases A_2_ have emerged as important players [13,15,30].

The role of extracellular vesicles in transcellular communication and especially in the pathophysiology of a wide spectrum of pro-inflammatory disorders, including cancer, is increasingly appreciated, [31,32]. In this frame, we investigated whether secretory phospholipase A_2_-IIA, (sPLA_2_-IIA), a pro-inflammatory marker, can be carried in the extracellular space by EVs. In this respect, EVs were isolated from BAL fluid of early- and late-phase ARDS and non-ARDS (control) patients and characterized. We found that: (1) BAL fluids from all the groups of patients with early and late ARDS and controls contained EVs bearing biochemical (e.g., CD63 protein marker) and morphological characteristics of exosomes, (2) *PLA2G2A* mRNA was present in all the EV preparations but most abundantly in early ARDS EVs, and, (3) sPLA_2_-IIA protein was detected in the small EV preparations only from patients with early ARDS.

Typical exosomal type EV isolation techniques include differential centrifugations, coupled to sucrose density gradients or even magnetic separations for further purification [33,34]. The morphological and biophysical characterization of the exosomes collected from biological fluids is still a challenging issue, especially when combined by phenotyping strategies to identify their cellular origin and cargo molecules.

We recovered a crude mixture of small EVs at the 120,000 g pellet of BAL fluid’s 40,000 g supernatants from early and late ARDS and non-ARDS (control) patients and fractionated them on a sucrose density gradient. By applying a variety of techniques, we showed that our preparation included EVs with the morphology, size/density range and biochemical characteristics of exosomes. Analysis of our crude EV preparations by DLS and TEM revealed heterogeneity in the morphology and size of vesicular structures. This is not any surprise, considering the biological complexity of BAL fluid and its enrichment in lung surfactant vesicular formations and aggregates [15,35]. In fact, cell-depleted BAL fluids from ARDS patients contained small surfactant vesicles, probably representing decomposed surfactant with poor surface properties. Some of this material may be recovered in the crude 120,000 g pellets. However, the surfactant vesicles are of low density and they are removed after purification on sucrose density gradient. The fractions corresponding to densities typical for EVs were positive for the tetraspanin CD63 and negative for the endoplasmic reticulum marker GRP-78. We did not observe any significant differences in the amount of the total vesicular protein recovered after the purification steps, or in the morphological characteristics of isolated exosomes from early-, late-ARDS and non-ARDS-control patients with the exception of extracellular multivesicular bodies detected in early ARDS samples but not in control samples. Extracellular multivesicular bodies, also termed matrix vesicle clusters/multivesicular cargo, are a peculiar EV type with the appearance of a multivesicular body, which consist of clusters of vesicles surrounded by a membrane. Although scarcely investigated, these extracellular vesicular structures have been previously detected in the interstitial spaces of vessel walls and between inflammatory and stromal cells in tissues performing inflammatory/repair processes [36]. Consequently, their detection in the EV pellet of BAL from early ARDS patients may further indicate the close relationship between vesicle release and the inflammatory response in ARDS including the increase in the permeability of the bronchoalveolar barrier. However, due to the complexity of the preparation steps, this point needs further validation.

Even though the role of EVs including exosomes in the lungs is still obscured, several observations have linked EVs with the development of lung injury/ARDS. Generation of microvesicles has been observed in platelets, neutrophils, monocytes, lymphocytes, red blood cells, as well as in endothelial and epithelial cells from patients with acute lung injury (ALI). Endothelial cell-derived EVs contain S1PR3, representing the inflammatory states of ALI, and are also believed to be important markers of lung vascular injury [37,38,39]. Τhey are reported to participate in immune surveillance or in the pathogenesis of lung disorders, for example, by potentiating or disseminating inflammation, [40,41]. Exosomal type EVs, recovered in BAL fluid from healthy individuals, contain MHC molecules that may regulate locally host defense [42]. In several pathological conditions, including sarcoidosis, the quantity of exosomes in BAL fluid is increased; they present a higher expression of MHC class-I and -II and other bioactive molecules, such as neuregulin-1 and they can activate autologous cells to produce inflammatory cytokines [43]. In asthma, BAL fluid EVs/exosomes display particular miRNA profiles [44] and carry enzymes for leukotriene biosynthesis [45]. BAL fluid exosomes might contribute to subclinical inflammation in asthmatics by increasing cytokine and leukotriene generation in the airway epithelium. In addition, in mouse models of ALI/ARDS, EVs derived from stressed epithelial cells activated macrophages to induce neutrophil infiltration, inflammatory cytokine bursts and lung inflammation [46].

Secretory PLA_2_-IIA is associated with the pathogenesis of ALI/ARDS [13,15]. In particular, it can deteriorate pulmonary function directly, by hydrolyzing lung surfactant phospholipids [14,47,48], or indirectly, through the production of potent lipid mediators, such as eicosanoids, platelet-activating factor or even excessive levels of lysophospholipids [19,49]. Our results support the occurrence of sPLA_2_-IIA in exosomal type EVs under two forms: *PLA2G2A* mRNA and protein. According to our best knowledge, the concurrent presence of both gene products in EVs has not been reported before. The fact that *PLA2G2A* mRNA is present in all exosomal types of EVs, but is more prominent in early ARDS, while the protein is detected only in the EVs with early phase ARDS, reveals a specific role of this enzyme in the dissemination of inflammation and suggests its probable use as an early diagnostic pro-inflammatory marker in ARDS.

This finding is consistent to our previous findings, according to which Ca^2+^-dependent sPLA_2_ types were localized in both the 100,000 g-pelleted particulate fraction of BAL fluid and in the 100,000 g supernatant [10,50]. Moreover, this supports the concept of sPLA_2_-IIA as an acute phase protein [8] that can justify its presence in the early and not in the late phase of ARDS. The underlying regulatory signals and the individual contribution of each sPLA_2_-IIA form to the progression and development of ARDS and other lung diseases can give an insight on the dissemination of inflammation, deserving further investigation. Of note, our findings agree with a study showing that different phospholipase activities, including PLA_2_s that participate in the production of proinflammatory lipid mediators, are recovered in EV/exosomes preparations from rat cell lines [51].

To our knowledge, this is the first description of the exosomal localization of a secreted PLA_2_ isoform in human samples. Exosomal sPLA_2_-IIA is probably involved in the sensitization of recipient cells in the lung during the development of ARDS and it is functionally distinct from soluble sPLA_2_ present in the BAL fluid, which is presumably implicated in lung surfactant decomposition. The differential expression of sPLA_2_-IIA mRNA and protein in exosome-type EVs released in the BAL fluid of early ARDS patients is of potentially significant diagnostic value. Moreover, it suggests a pathophysiological role of sPLA_2_ in the dissemination of the inflammatory response and the disturbed permeability of bronchoalveolar barrier which characterizes the exudative phase of ARDS. By pointing out new therapeutic targeting of the inflammatory arm of ARDS, the present data deserve further study through state-of-the-art nanotechnological strategies in larger cohorts of mechanically ventilated patients with/without ARDS.

## 4. Materials and Methods

### 4.1. Patients

Twenty-one mechanically ventilated patients were included in this study; nine of them suffered from early ARDS, five from late ARDS and seven served as a non-ARDS control group. Standard criteria for ARDS diagnosis were based on the Berlin definition, namely the acute, diffuse, inflammatory lung injury accompanied with hypoxemia and bilateral opacities on chest radiograph or computed tomography scan not fully explained by cardiac failure of fluid overload [52]. The causes of ARDS were both primary (pneumonia, aspiration of gastric content and lung contusion) and secondary (severe sepsis due to catheter-related infections, abdominal sepsis and pancreatitis). The ARDS patients were grouped into early or late phase, according to the clinical course, as defined below. The non-ARDS control group included hemodynamically stable mechanically ventilated patients, suffering from neuromuscular diseases, with normal chest radiograph, who developed ventilator failure and PaO_2_/FiO_2_ >300 mmHg, but without evidence of cardiopulmonary disease or systemic inflammation. The study was conducted in accordance with the Declaration of Helsinki and the protocol was approved by the review board for human studies of the University Hospital of Ioannina. The patients or the next of kin gave written informed consent for inclusion before they participated in the study.

### 4.2. Bronchoalveolar Lavage

Bronchoalveolar lavage (BAL) was performed by fiberoptic bronchoscopy, when the patients were hemodynamically stable, within 24 h from the diagnosis of ARDS for early phase ARDS, or for more than 6 days for late phase of ARDS. In summary, all the patients were ventilated with a controlled mechanical ventilation mode, as previously reported [15]. Six aliquots of 20 mL of sterile normal saline at 37 °C were infused through the working channel of the bronchoscope. The first aspirated fluid, reflecting a bronchial sample, was discarded, whereas the others were collected in the same ice-cold container and filtered through 70 m nylon cell-strainer filters (Falcon, Becton Dickinson, Franklin Lakes, NJ, USA) to remove mucus. The BAL fluid was obtained after a centrifugation step of the filtrate at 800 g for 20 min at 4 °C, during which BAL cells were removed. The BAL fluid was used for diagnostic tests, biochemical analyses and the isolation of EVs.

### 4.3. Isolation and Purification of EVs

All the procedures were carried out at 4 °C. Extracellular vesicles, including exosomes, were isolated from approximately 60 mL of fresh BAL fluid preparations through differential centrifugations. The 800 g supernatants, after cell removal, were centrifuged at 15,000 g for 30 min to remove cell debris, and the 15,000 g supernatant was centrifuged at 40,000 g for 1 h to remove large aggregates of surfactant and large EVs (of microvesicles type). The final spin was performed at 120,000 g for 1 h, using a Beckman L7 ultracentrifuge, to isolate the pellet composed of small EVs, including exosomes (crude small EVs’ fractions).

Fractionation of the crude EVs’ fractions was performed on a continuous sucrose gradient: The 120,000 g pellet preparations were resuspended in 1 mL of 2.5 M sucrose/HEPES solution, laid over a continuous sucrose gradient (0.25–2.0 M sucrose, 20 mM HEPES/NaOH pH 7.4) and ultracentrifuged at 120,000 g for 15–20 h at 4 °C, using a swing-out SW-41 Beckman rotor. One milliliter gradient fractions were collected from the top of the tube. Fraction densities were examined by a Wya Abbe refractometer. Each density fraction was resuspended into 10 mL PBS, centrifuged at 120,000 g for 1 h to pellet vesicles and, finally, the pellets were resuspended into 100–200 μL PBS and kept at 80 °C until use.

### 4.4. Dynamic Light Scattering

One milliliter fractions from the EV preparations were collected after ultracentrifugation on a continuous sucrose gradient and analyzed with a Zetasizer ZS (Malvern Instruments, Worcestershire, UK). Temperature was set at 25 °C and they were allowed to equilibrate for several minutes. Parameters of zeta potential and intensity-, volume- and number-weighted size distributions were measured.

### 4.5. Total RNA Isolation and qRT-PCR

For qRT-PCR experiments, equal protein amounts of EVs isolated from the BAL fluid of patients with early and late phase ARDS and non-ARDS controls, and further purified with sucrose gradient, as described before, were firstly lysed with the addition of lysis buffer solution provided by the NucleoSpin RNA II kit (Macherey-Nagel, GmbH & Co. KG, Düren, Germany). Total RNA was isolated according to the manufacturer’s recommended protocol. RNA purity was verified photometrically with the criterion of OD_260_/OD_280_ absorption ratio greater than 1.7. BAL fluids from non-ARDS patients were used as control samples. Real-time PCR was performed using the iScript One-Step RT-PCR kit with SYBR green (Bio-Rad Laboratories, Hercules, CA, USA), using forward and reverse primers from Qiagen (Germantown, MD, USA) for *PLA2G2A* and *GAPDH*, with the second used as a reference housekeeping gene. Total RNA (20 ng) in a 20 μL total volume was firstly incubated at 50 °C for 10 min, for the action of the reverse transcriptase and cDNA synthesis, heated at 95 °C for the dilation of double stranded molecules and the denaturation of reverse transcriptase and then subjected to 35 thermal cycles (94 °C for 40 s, 60 °C for 40 s, 72 °C for 1 min) for PCR amplification and 40 cycles from 55 °C to 95 °C (1 °C increase/cycle) for melting curve analysis using MJ mini thermal cycler (Bio-Rad Laboratories, Hercules, CA, USA). Quantitative RT-PCR results were calculated as fold-increase in gene mRNA versus fold-increase in *GAPDH* mRNA. Data from qRT-PCR from BAL fluid EVs RNA samples were recorded and calculated with Bio-Rad CFX Manager software applying the ΔΔCt method.

### 4.6. Western Blotting

Exosomal EV preparations (1 μg protein, as quantified by the Bradford assay) were denatured in Laemmli buffer and by boiling for 5 min. For better resolution of proteins on SDS polyacrylamide gels, the following acrylamide concentrations were used: 10% for Grp-78, 12% for CD63 and 15% for sPLA_2_-IIA. Following transfer onto PVDF membranes (Laemmli, 1970), they were incubated with blocking solution (5% non-fat milk in TBS-Tween 20) overnight at 4°C and then incubated with the appropriate dilutions of primary antibodies in PBS (overnight at 4 °C) as follows: CD63 (E-12) (sc-365604, Santa Cruz Biotechnology, Inc., Santa Cruz, CA, USA, mouse monoclonal, dilution 1:1000), sPLA_2_ group IIA (ab23705, ABCAM, Cambridge, MA, USA, polyclonal rabbit anti-human, dilution 1:1000) and Grp-78 (H-1296) (sc-13968, Santa Cruz Biotechnology, Inc., CA, USA, dilution 1:1000). After washing with TBS-T, blots were incubated for 2 h at room temperature with the appropriate secondary antibodies: goat anti-rabbit IgG-HRP antibody (sc-2004, Santa Cruz Biotechnology, Santa Cruz, CA, USA, dilution 1:10,000 in TBS-Tween 20) and goat anti-mouse IgG-HRP, (sc-2005, Santa Cruz Biotechnology, Inc., Santa Cruz, CA, USA, dilution 1:10,000 in TBS-Tween 20). The immunoreactive bands were visualized with Luminata Crescendo Western HRP Substrate, WBLURO100 (Millipore, Temecula, CA, USA).

### 4.7. Confocal Microscopy

EV preparations were incubated on poly-L-lysine coated coverslips for 15 min, washed with PBS and then fixed with 4% paraformaldehyde (PFA) at room temperature, for 20 min. After washing with PBS and permeabilization with 0.01% saponin in PBS, attached vesicles were incubated with anti-CD63 and anti-sPLA_2_-IIA mAb overnight, at 4 °C, after blocking with 3% BSA in PBS. Following further washing, samples were incubated with Alexa Fluor 488, and Alexa Fluor 568-conjugated secondary antibodies and visualized by a Leica SP5 confocal microscope applying standard settings for the above fluorescent dyes.

### 4.8. Transmission Electron Microscopy

Conventional transmission electron microscopic (TEM) analysis was performed on freshly isolated EV pellets as follows: Immediately after the final centrifugation step, the pellets derived from approximately 60 mL of BAL fluid were resuspended into 50–100 μL of PBS (pH 7.4) containing 4% methanol-free paraformaldehyde (Polysciences, Inc., Warrington, PA, USA) in PBS. EV suspensions were carefully applied in 5 μL drop portions on formvar-carbon coated grids (Electron Microscopy Sciences, Hatfield, PA, USA) and allowed to absorb for 10 min. Grids bearing the vesicles were rinsed with 50 mM glycine in PBS, fixed with 1% glutaraldehyde in PBS, for 5 min at room temperature, stained with 2% osmium tetroxide water solution, washed and examined by a JEOL 2100 Transmission Electron Microscope at 200 kV accelerating voltage.

For immuno-gold electron microscopy, a similar protocol was followed. Briefly, grids with the absorbed EV samples were mildly permeabilized with saponin solution in PBS and then placed on drops of a mix of primary antibodies (mouse anti-CD63 at a working dilution of 1/100 and rabbit anti-sPLA_2_-IIA at a working dilution of 1/50) in PBS containing 3% BSA for 30 min at room temperature. Following washing with PBS/3% BSA (5 × 3 min), the grids were incubated with mixed (5 and 15 nm) gold-conjugated probe solution (anti-mouse IgGs, 5 nm gold 1/50 and anti-rabbit-IgGs 15 nm gold 1/70 (British Biocell International, Cardiff, UK) in PBS/3% BSA for 30 min, washed with 100 μL drops of PBS/3% BSA (5 × 3 min) and stained with uranyl acetate before visualization by TEM. Routine procedures were applied as controls (i.e., omission of the primary Ab, use of an irrelevant control primary Ab) to demonstrate the specificity of the immune reaction. For single staining of CD63 exosome marker, membrane permeabilization step was omitted.

## 5. Conclusions

The BAL fluid from mechanically ventilated patients with/without ARDS contains EVs with biochemical and morphological characteristics relevant to “exosomes”. sPLA_2_-IIA mRNA is a component of these exosomes and further shows substantially higher expression in patients with early ARDS compared to both late ARDS and non-ARDS individuals. In addition, the sPLA_2_-IIA protein is selectively detected in the acute phase ARDS EVs, suggesting that vesicular sPLA_2_-IIA can be a useful marker in the early diagnosis of the syndrome. The prominent co-localization of sPLA_2_-IIA at both protein and mRNA levels on exosomal-like EVs in early ARDS, highlights its role in the dissemination of the immediate and longer-term inflammatory response. A dual potential route of action, both locally by promoting hydrolytic reactions and production of bioactive lipids in the extracellular lung environment and at distance by changing the immune status of outlying recipient cells, renders sPLA_2_-IIA a multifaceted biological response modifier critically involved in the pathophysiology of ARDS and, thus, in its diagnosis and therapeutic targeting in the future.

## Figures and Tables

**Table 1:** Dynamic light scattering (DLS) analysis of vesicles in density fractions of bronchoalveolar lavage (BAL) fluid.

Sample Fractions	Density (g/mL)	Mean Diameter (nm) *	Z-ave (nm) ^†^	PdI ^‡^
1	1.09	339.50 ± 24.52	379.90 ± 17.24	0.253 ± 0.154
2	1.11	55.70 ± 7.14	361.60 ± 23.41	0.582 ± 0.147
3	1.13	26.65 ± 2.30	249.80 ± 3.81	0.573 ± 0.120
4	1.14	31.38 ± 3.95	313.20 ± 16.60	0.484 ± 0.070
5	1.15	46.37 ± 9.02	299.55 ± 3.46	0.663 ± 0.240
6	1.17	51.51 ± 1.25	269.83 ± 39.26	0.504 ± 0.263
7	1.20	162.30 ± 12.30	233.56 ± 28.24	0.513 ± 0.134
8	1.22	103.52 ± 5.64	195.65 ± 12.65	0.605 ± 0.036

* Mean diameter, numbers represent mean value ± standard error (*n* = 3 measurements per fraction) from a representative density gradient separation of Bronchoalveolar Lavage Fluid from an acute respiratory distress syndrome (ARDS) patient. ^†^ Z-ave, Z-average. ^‡^ PdI, Polydispersity Index.
